# Modeling Risk Factors for Intraindividual Variability: A Mixed-Effects Beta-Binomial Model Applied to Cognitive Function in Older People in the English Longitudinal Study of Ageing

**DOI:** 10.1093/aje/kwad169

**Published:** 2023-08-11

**Authors:** Richard M A Parker, Kate Tilling, Graciela Muniz Terrera, Jessica K Barrett

**Keywords:** Bayesian hierarchical model, beta-binomial model, cognitive tests, heteroscedasticity, intraindividual variability, mixed-effects model, older adults

## Abstract

Cognitive functioning in older age profoundly impacts quality of life and health. While most research on cognition in older age has focused on mean levels, intraindividual variability (IIV) around this may have risk factors and outcomes independent of the mean value. Investigating risk factors associated with IIV has typically involved deriving a summary statistic for each person from residual error around a fitted mean. However, this ignores uncertainty in the estimates, prohibits exploring associations with time-varying factors, and is biased by floor/ceiling effects. To address this, we propose a mixed-effects location scale beta-binomial model for estimating average probability and IIV in a word recall test in the English Longitudinal Study of Ageing. After adjusting for mean performance, an analysis of 9,873 individuals across 7 (mean = 3.4) waves (2002–2015) found IIV to be greater at older ages, with lower education, in females, with more difficulties in activities of daily living, in later birth cohorts, and when interviewers recorded issues potentially affecting test performance. Our study introduces a novel method for identifying groups with greater IIV in bounded discrete outcomes. Our findings have implications for daily functioning and care, and further work is needed to identify the impact for future health outcomes.

## Abbreviations

ADLs: activities of daily living
CrI: credible interval
ELSA: English Longitudinal Study of Ageing
HIV: human immunodeficiency virus
IIV: intraindividual variability
MELS: mixed-effects location scale
OR: odds ratio

Cognitive functioning in older people has profound implications for current and future health and well-being (1, 2). In the absence of therapeutic cures for dementia, for example, changes in cognitive performance can aid in the early identification of individuals at increased risk of developing the condition, which is paramount for the design and implementation of interventions that may delay the onset of faster deterioration (3, 4). Traditionally, research in cognitive decline has focused on the study of individual differences and the identification of risk factors for rate of mean change (5). However, some researchers have investigated inconsistency in performance (6, 7). For example, in an analysis of data from the Sydney Memory and Aging Study, Kochan et al. (8) found that greater intraindividual variability (IIV), but not greater mean reaction time, significantly predicted survival time after adjustment for sociodemographic factors, cardiovascular disease risk, and apolipoprotein E ε4 allele status. Similarly, Gamaldo et al. (9) investigated data from the Baltimore Longitudinal Study of Aging, showing that individuals who had received a diagnosis of dementia had greater variability in attention, executive function, language, and semantic memory at least 5 years before the onset of cognitive impairment than individuals who remained free of dementia, demonstrating the potential role of IIV as an early indicator of pathological changes.

Despite the increasing interest in IIV, the analytical approaches commonly used to quantify it in longitudinal studies are limited. Some researchers have considered the average amount of deviation (residual error around a fitted mean) in an individual’s performance over time (10, 11). However, this does not adjust for uncertainty in the estimates given the finite number of within-person observations, and the resulting individual-level summary statistic is not amenable to the exploration of associations of IIV with time-varying factors. Alternatively, Gamaldo et al. fitted multilevel models to repeated measurements of the outcome of interest, and then compared models which assume the residual IIV to be constant with models which allow it to depend on fixed effects (e.g., diagnostic status) (9). However, this assumes that people within each group have the same IIV. It has been previously shown that in a multilevel model, the residual IIV can instead be assumed to contain systematic variation that can be explained, depending not only on fixed effects (as in Gamaldo et al. (9)) but on random effects as well, in mixed-effects location scale (MELS) models (12–16). MELS models allow the associations of IIV with predictors which may be time-varying, or otherwise, to be investigated. They further allow for residual differences between people in their IIV to be estimated via random effects, and their association with the individual mean to be investigated via correlated random effects (12).

While MELS models typically assume that, conditional on the random effects, the response variable is normally distributed, this is likely to be violated with bounded discrete outcomes, which are commonly used in the assessment of a variety of domains (17, 18). Here, floor and ceiling effects can lead to underestimated IIV for people returning high or low mean scores. Under such circumstances, a beta-binomial model has been shown to improve statistical inference (18). In contrast to some other approaches to analyzing such data, including Gaussian, Poisson, and negative binomial models, the beta-binomial model supports nonnegative integers with an upper bound—qualities found in many cognitive test outcomes. In addition, rather than directly modeling the probability of trial success, it models the distribution of probabilities. To investigate factors associated with IIV in cognitive performance, we propose a model somewhat analogous to the MELS formulation, where the “location” and “scale” parameters of the beta-binomial model are each allowed to differ across fixed and random effects.

Since the evidence on factors associated with differences in IIV over time is limited, we used a MELS beta-binomial model to investigate visit-to-visit IIV in a word recall test in the English Longitudinal Study of Ageing (ELSA). Our aim was to introduce a modeling approach appropriate to this bounded discrete outcome to better understand the factors associated with IIV in a test of episodic memory in older adults.

## METHODS

### Cohort

Participants were from ELSA, an ongoing panel study that contains a nationally representative sample of the English population aged 50 years and over living in households (19, 20). Interviews were carried out with 11,391 individuals (5,186 men and 6,205 women) at baseline (2002–2003); the overall response rate was 70% at the household level and 67% at the individual level. After the baseline interview, follow-up interviews took place at regular 2-year intervals in 2004–2005 (wave 2), 2006–2007 (wave 3), 2008–2009 (wave 4), 2010–2011 (wave 5), 2012–2013 (wave 6), and 2014–2015 (wave 7). Refresher samples were added at waves 3, 4, 6, and 7 to ensure that the study remained representative of the target age group. Participants gave full informed consent to participate in the study. We restricted our sample to core participants and to observations made when aged 65 years or older for which the participant did not report having Alzheimer disease or dementia, organic brain syndrome, senility, or any other serious memory impairment.

### Cognitive function

Memory was measured using a 10-word recall test, as earlier used in the Health and Retirement Study (21). Participants were presented with a list of 10 words that were read out to them and asked to recall as many words as they could both immediately and, with no prior notice, 5 minutes later and after they had been asked to complete other survey questions. The number of correctly recalled words was used as a measure of memory (range, 0–20 words), adding the results from both the immediate and delayed recall tests.

### Covariates

Information on participants’ age, sex, education, and difficulties with activities of daily living (ADLs) was recorded at each wave. In addition, the interviewer reported whether there were any factors which may have impaired the participant’s performance during the cognitive tests. Age was centered (on the sample mean of 74.2 years) and in decades. Cohort was designated as the year in which the participant turned 65 years of age, centered around 1999, also as decades. Education was categorized into higher education (college/university), secondary education, and no UK educational qualifications. For difficulties with ADLs, participants were asked whether they had any difficulty dressing (including putting on shoes and socks), eating (including cutting up food), bathing and showering, getting into and out of bed, or walking across a room; a count of the number of items participants had difficulties with was derived from this. Interviewer-recorded factors which may have affected the cognitive tests included the participant’s being blind or having poor eyesight, being deaf or having poor hearing, being too tired, having illness or physical impairment, having impaired concentration, being very nervous or anxious, or having another mental impairment; an interruption or distraction; a noisy environment; problems with the testing computer; difficulty in understanding English; or any other factor. The resulting variable took the value of 0 if there were no such issues recorded and 1 otherwise. Difficulties with ADLs and interviewer-recorded factors potentially affecting the tests were both fitted as person-level mean values, to estimate their between-individual effect, and as person-level mean-centered variables, to estimate their within-individual effect (22).

### Analytical approach

A MELS beta-binomial model was fitted to repeated measurements of the longitudinal outcome, the word recall test score. The beta-binomial model assumes that each observed value of the outcome is the result of *n* Bernoulli trials which have an underlying, unobserved probability of success as sampled from a beta distribution (23, 24). The shape of the beta distribution from which these probabilities are drawn is defined by an average probability parameter *p* (i.e., μ; modeled on the logit scale) and a variability (or dispersion or variance) parameter θ (i.e., φ, κ, or ρ; modeled on the log scale) (17, 25, 26).

**Figure 1:**
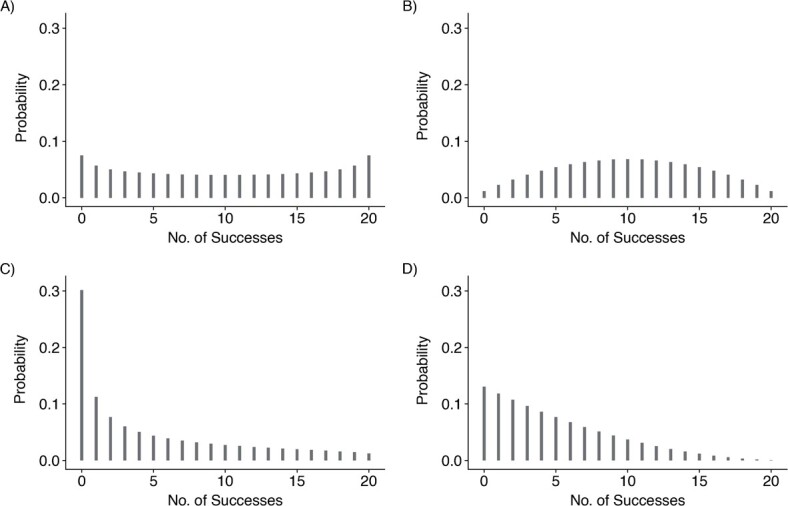
Expected probabilities of scores from a test consisting of 20 trials (as in the test of word recall used in the English Longitudinal Study of Ageing (2002–2015)), assuming beta distributions of underlying probabilities with different values of average probability (*p*) and dispersion (θ), as follows: A) *p* = 0.5, θ = 1.5; B) *p* = 0.5, θ = 4.0; C) *p* = 0.2, θ = 1.5; D) *p* = 0.2, θ = 4.0. For example, when *p* = 0.5, if θ > 2 (e.g., panel B), the distribution of scores is relatively concentrated around the mean score of 10, while if θ < 2 (e.g., panel A) dispersion is greater and extreme scores near 0 and 20 are more likely than the mean (if θ is 2 (not shown), then each possible score, from 0 to 20, would be equally likely (25)). As this figure further illustrates, the value of θ is orthogonal to *p*. See Web Appendix 3 and Web Figure 2 for additional examples.

Instead of directly modeling the probability for each observed count, the beta-binomial models this distribution of probabilities, via *p* and θ. While a MELS model is typically a Gaussian model, we use the terminology here because we include both fixed (population) and random (individual) effects in 1) the linear predictor for *p* (the “location” of the beta distribution) and 2) the linear predictor for θ (the “scale” of the beta distribution, where low estimated values of θ imply greater IIV in task performance). Figure 1 plots the expected distributions of test scores given illustrative values for these parameters.

Covariates added to the linear predictor for *p* were age, cohort (the year of reaching age 65), sex, educational qualifications, the number of ADLs with which the respondent reported difficulty, and whether the interviewer reported any factors which may have impaired the participant’s performance during the cognitive tests. These covariates were also included in the linear predictor for θ. For the linear predictor for *p*, any nonlinearity in the association between age and the outcome was first assessed by fitting restricted cubic regression splines with different sets of knots as recommended by Harrell (27), and the best-fitting function of age was selected and fitted. Interactions of age with each of sex, educational qualifications, number of ADL difficulties, and issues potentially impairing test performance were also added, in turn, to the linear predictor for *p* to see whether they improved model fit. Model fit was assessed via Pareto smoothed importance sampling leave-one-out (PSIS-LOO) cross-validation (28). In addition, random effects were included in the functions for both *p* and θ to account for unobserved heterogeneity between individuals. In the predictor for *p*, a random intercept estimated the between-individual variability in *p* at the mean age, and a random slope estimated the between-individual variability of the effect of age (as a linear term fitted across the whole age range) on *p*. In the linear predictor for θ, a random intercept estimated the extent to which people differed in their IIV (specifically in how dispersed the beta distribution was from which the underlying probability of test success was drawn; see Figure 1 for examples). Random effects were assumed to be multivariate normally distributed, allowing for nonzero correlations between them.

The models were fitted using Bayesian estimation via Markov chain Monte Carlo methods in Stan (version 2.21.0), using the “brms” package (version 2.16.1) in R, version 4.1.0 (R Foundation for Statistical Computing, Vienna, Austria) (29–31). Results are reported as means of posterior distributions and 95% credible intervals (CrIs). See Web Appendices 1 and 2 (available at https://doi.org/10.1093/aje/kwad169) for further details on these (and other sensitivity) analyses, including choice of priors, and sample code.

### Ethics approval

ELSA was conducted in accordance with the Declaration of Helsinki, and ethical approval was granted by the National Research Ethics Service (London Multicentre Research Ethics Committee).

## RESULTS

A total of 9,873 individuals were included in the final cohort (see Web Figure 1). Of these, 2,202 participants (22.3%) were reported as dying during the study period, while the mortality status of 2,477 participants (25.1%) was reported as unknown at the final wave (wave 7), with the remaining 5,194 (52.6%) being reported as alive at that wave. Of persons in the final cohort, 396 (4.0%) were reported as having a memory problem in at least 1 survey (these and subsequent surveys for such participants were not included in the model). On average, participants included in the final cohort contributed data to 3.4 waves, with the number of observations per participant as follows: wave 1, 2,313; wave 2, 1,866; wave 3, 1,425; wave 4, 1,470; wave 5, 751; wave 6, 800; and wave 7, 1,248.

Table 1 shows the baseline characteristics of included individuals, including by whether or not they contributed data to every wave after becoming eligible. It indicates that those who did not contribute data to every wave had a lower memory test score, were older, had lower educational qualifications, had more difficulties with ADLs, and had more issues recorded by the interviewer which may have affected the cognitive tests, on average.

**Table 1:** Baseline Characteristics of Individuals Included in the Analytical Model Fitted to Investigate Intraindividual Variability in Word Recall Test Performance, According to Whether or Not They Contributed Data to Every Study Wave After Becoming Eligible for Inclusion, English Longitudinal Study of Ageing, 2002–2015

**Baseline Characteristic** ^**a**^	**Total** **(*n* = 9,873)**		**Did Not Contribute** **Data to Every Wave** **(*n* = 5,421)**		**Contributed Data** **to Every Wave** **(*n* = 4,452)**	
	**No.**	**%**	**No.**	**%**	**No.**	**%**
Age, years^b^^,^^c^	70.5 (6.4)		73.1 (7.0)		67.3 (3.5)	
Cohort^d^						
1971–1980	282	2.9	278	5.1	4	0.1
1981–1990	1,595	16.2	1,444	26.6	151	3.4
1991–2000	3,058	31.0	2,098	38.7	960	21.6
2001–2010	3,574	36.2	1,488	27.4	2,086	46.9
2011–2020	1,364	13.8	113	2.1	1,251	28.1
Sex						
Male	4,490	45.5	2,512	46.3	1,978	44.4
Female	5,383	54.5	2,909	53.7	2,474	55.6
Education						
None	4,731	47.9	3,223	59.5	1,508	33.9
Secondary	2,849	28.9	1,327	24.5	1,522	34.2
Higher education	2,293	23.2	871	16.1	1,422	31.9
No. of ADLs performed with difficulty^e^						
0	7,723	78.2	3,912	72.2	3,811	85.6
1	1,158	11.7	783	14.4	375	8.4
2	521	5.3	361	6.7	160	3.6
3	292	3.0	216	4.0	76	1.7
4	144	1.5	120	2.2	24	0.5
5	35	0.4	29	0.5	6	0.1
Issues with cognitive tests^f^						
No	8,634	87.5	4,515	83.3	4,119	92.5
Yes	1,239	12.5	906	16.7	333	7.5
Word recall test score^b^	9.2 (3.7)		7.9 (3.6)		10.7 (3.2)	

Abbreviation: ADLs, activities of daily living.

^a^ “Baseline” was the first wave of inclusion in the model for each individual.

^b^ Values are expressed as mean (standard deviation).

^c^ Note that this differs from the sample mean quoted in the main text, as it is calculated from the baseline observations only, rather than from all of the repeated measurements.

^d^ Year in which the participant turned age 65 years (presented as an ordinal variable here, but included in the analytical models as a continuous variable).

^e^ Sum of the number of ADLs with which the participant reported having any difficulty. ADLs include bathing, dressing, eating, getting into/out of bed, and walking across a room.

^f^ Interviewers recorded any issues which may have affected the participant’s performance on the cognitive tests.

**Figure 2:**
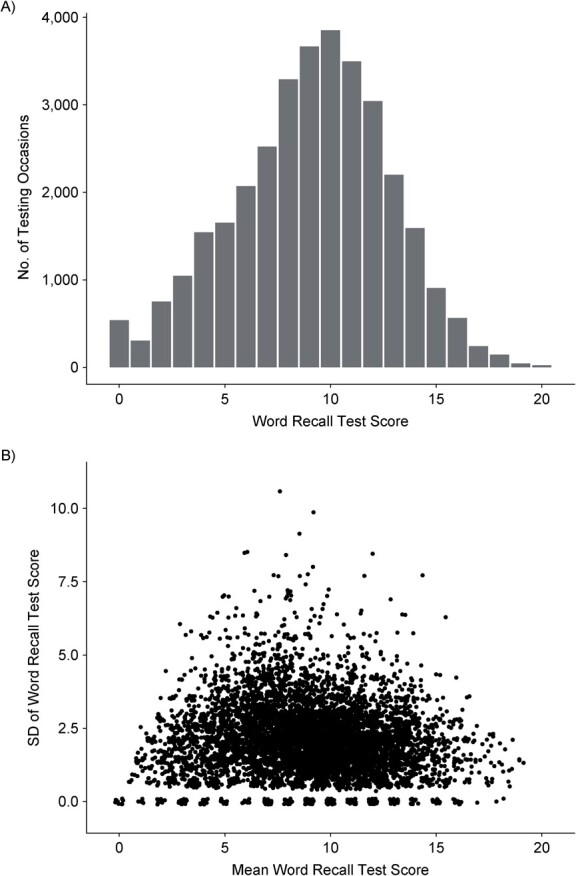
Word recall test scores (A) and plots of standard deviations (SDs) of word recall test scores against mean test scores (B) in the English Longitudinal Study of Ageing, 2002–2015. The maximum possible score was 20. In panel B, only data from participants observed on 2 or more occasions are plotted (to calculate SD), with a small amount of added jitter to avoid overplotting.

Figure 2 plots the distribution of word recall test scores (Figure 2A) and the mean score versus the standard deviation (Figure 2B), with the standard deviation generally being lower for people with high or low mean scores.

See Web Tables 1–4 for further summary statistics, including results presented by exclusion status and dropout status, and also for all estimates from the models presented below.

### Association of covariates with *p* (average probability)

**Figure 3:**
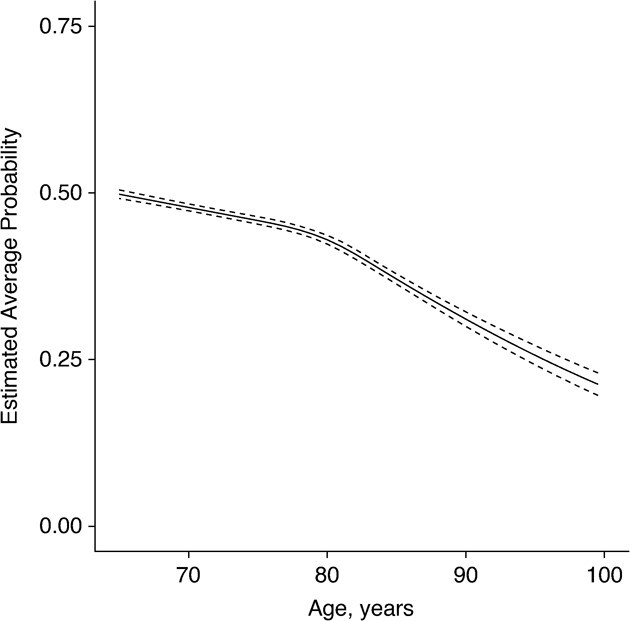
Predicted average probability (*p*) of correctly recalling each item in the word recall test, by age, English Longitudinal Study of Ageing, 2002–2015. Dashed lines show the 95% credible intervals.

A restricted cubic spline for age, with 4 knot points, was fitted in the fixed part of the linear model for *p*; no interaction terms were selected (see Web Appendix 1). Figure 3 presents the estimated average probability of providing a correct answer in the word recall test, across age. It indicates that, on average, this probability decreased with age, with the rate of this decline being greater from around 80 years of age onward.

**Figure 4:**
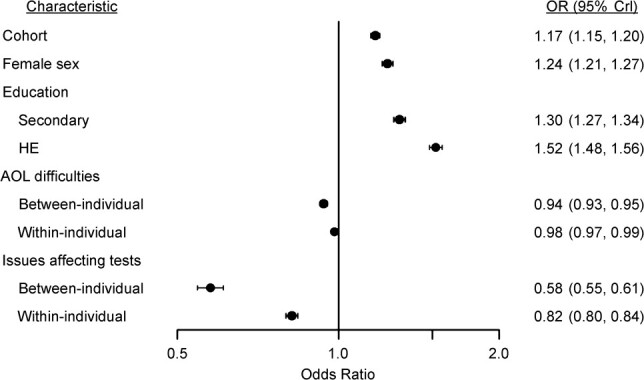
Estimated odds ratios (ORs) for characteristics modeled in the linear predictor for average probability (*p*) in the word recall test (WRT), English Longitudinal Study of Ageing, 2002–2015. For “Cohort,” the OR is the estimated average OR for recalling a word correctly on the WRT per 1-decade change in birth cohort (the year in which the participant turned 65 years of age; centered on 1999). For “Female sex” and “Education,” the OR is the estimated average OR for recalling a word correctly on the WRT when the current category was compared with the respective reference category (male sex or no qualifications). For “ADL difficulties,” the OR represents the estimated average odds of recalling a word correctly on the WRT for each additional ADL difficulty reported, fitted as a person-level mean to estimate the between-individual effect and also as a person-level mean-centered variable to estimate the within-individual effect (22). For “Issues affecting tests,” the OR is the estimated average OR for recalling a word correctly on the WRT when the current category (at least 1 interviewer-recorded factor which may have affected the test, coded as 1) was compared with the reference category (no such interviewer-recorded factors, coded as 0), fitted as a person-level mean to estimate the between-individual effect and also as a person-level mean-centered variable to estimate the within-individual effect (22). Bars show the 95% credible intervals (CrIs). ADL, activities of daily living; HE, higher education.

Figure 4 presents the estimated odds ratios (OR) for the remaining covariates in the linear predictor for *p*. It indicates the probability of correctly recalling words declined, on average, as the number of difficulties with ADLs increased, with an OR for the between-individual effect of 0.94 (95% CrI: 0.93, 0.95), indicating 6% lower odds of recalling a word correctly for each activity reported to be performed with difficulty, and an OR for the within-individual effect of 0.98 (95% CrI: 0.97, 0.99). The mean test score was lower when the interviewer recorded issues potentially affecting the test, with an OR for the between-individual effect of 0.58 (95% CrI: 0.55, 0.61) and an OR of 0.82 (95% CrI: 0.80, 0.84) for the within-individual effect. For sex, an estimated OR of 1.24 (95% CrI: 1.21, 1.27) indicated that the odds of recalling a word correctly were 24% higher for females than for males. The odds were also higher in those with a higher level of education: Compared with no educational qualifications, participants with secondary educational qualifications had an OR of 1.30 (95% CrI: 1.27, 1.34), and those with higher education qualifications had an OR of 1.52 (95% CrI: 1.48, 1.56). Finally, the odds were higher in later birth cohorts, with an OR of 1.17 (95% CrI: 1.15, 1.20) per decade of later birth.

### Association of covariates with θ (IIV)

**Figure 5:**
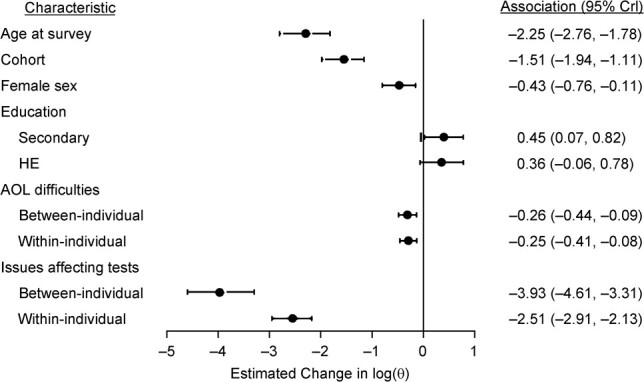
Estimated associations of modeled characteristics with the intraindividual variability parameter log(θ) in the word recall test, English Longitudinal Study of Ageing, 2002–2015. Smaller values of log(θ) indicate greater dispersion (intraindividual variability). For “Age at survey,” the association reflects the estimated change in log(θ) per 1-decade change in age (centered on the sample mean age of 74.2 years). For “Cohort,” the association reflects the estimated change in log(θ) per 1-decade change in birth cohort (the year in which the participant turned 65 years of age; centered on 1999). For “Female sex” and “Education,” the association reflects the estimated change in log(θ) when the current category was compared with the respective reference category (male sex or no qualifications). For “ADL difficulties,” the association reflects the estimated change in log(θ) for each additional ADL difficulty reported, fitted as a person-level mean to estimate the between-individual effect and also as a person-level mean-centered variable to estimate the within-individual effect (22). For “Issues affecting tests,” the association reflects the estimated change in log(θ) when the current category (at least 1 interviewer-recorded factor which may have affected the test, coded as 1) was compared with the reference category (no such interviewer-recorded factors, coded as 0), fitted as a person-level mean to estimate the between-individual effect and also as a person-level mean-centered variable to estimate the within-individual effect (22). Bars show the 95% credible intervals (CrIs). ADL, activities of daily living; HE, higher education.

Figure 5 presents the estimated change in the log of the IIV or dispersion parameter, θ, for each modeled characteristic (n.b. lower values of θ indicate greater IIV (e.g., Figure 1)). It indicates that older people had greater IIV in memory scores, on average, with log(θ) estimated to change by −2.25 (95% CrI: −2.76, −1.78) for each decade of increase in age at survey. As Figure 5 also illustrates, higher educational qualifications were associated with lower IIV; those who completed secondary school, for instance, had an estimated log(θ) that was higher by 0.45 (95% CrI: 0.07, 0.82) than individuals without qualifications. IIV was greater for individuals with more difficulties in their ADLs, with the between-individual effect estimated to change log(θ) by an average of −0.26 (95% CrI: −0.44, −0.09) for each activity performed with difficulty; the estimate for the within-individual effect was similar (−0.25, 95% CrI: −0.41, −0.08). IIV was higher for females, with a log(θ) estimated to differ from that of males by an average of −0.43 (95% CrI: −0.76, −0.11). When the interviewer had recorded issues which might have affected test performance, the IIV was higher, with log(θ) differing by an average of −3.93 (95% CrI: −4.61, −3.31) for the between-individual effect and by −2.51 (95% CrI: −2.91, −2.13) for the within-individual effect, as compared with situations where no such issues were recorded by the interviewer. Later birth cohorts were also estimated to have greater IIV, with log(θ) changing by −1.51 (95% CrI: −1.94, −1.11) for each decade of later birth.

### Participant effects

The random part of the model indicated that the correlation between average probability (*p*) at 74.2 years of age (random intercept) and rate of change in *p* (random slope) was estimated at 0.37 (95% CrI: 0.29, 0.47), suggesting that individuals with poorer episodic memory at age 74.2 years experienced a faster rate of decline in memory (Web Table 4). Those who tended to score higher on the memory test (a more positive random intercept for *p*) tended to have lower IIV (higher log(θ)), with a posterior mean correlation of 0.45 (95% CrI: 0.37, 0.52) between the random intercept (at 74.2 years of age) for *p* and the random intercept of the predictor for log(θ). Finally, the posterior mean correlation between the random slope for *p* and the random intercept for IIV (log(θ)) was 0.31 (95% CrI: 0.11, 0.51), indicating that persons with slopes which declined less steeply (i.e., more positive estimates for the random slope) tended to have lower IIV (i.e., a higher estimate for log(θ)).

## DISCUSSION

We have proposed and applied a MELS beta-binomial model to simultaneously estimate average probability and IIV in a discrete outcome, estimating fixed (population) and random (individual) effects for each. We found that IIV in a visit-to-visit test of episodic memory in older adults participating in ELSA changed across age, sex, education, number of ADL difficulties, cohort, and reported challenges when performing the test. We also found that people with a higher probability of recalling words correctly tended to have lower IIV, and those with a more gradual decline in mean performance over time also tended to have lower IIV.

Our finding that IIV in the word recall test increased with age, while the probability of correctly recalling words decreased with age, is characteristic of studies of cognitive functioning in advanced years—as reviewed by MacDonald et al. (7), for example, who further discussed potential neural mechanisms underlying these age-related changes in IIV. In addition, in earlier analyses of the ELSA cohort, investigators similarly found a faster average rate of decline in average word recall test performance at older ages (32, 33). Higher mean memory performance in later cohorts, as we found, has also previously been reported in ELSA (33), although we additionally found IIV to be greater in later cohorts as well.

Our results also indicated that the greater the number of difficulties participants had with ADLs, the greater their IIV in memory test performance and the lower their probability of correctly recalling words (with the between-individual effect being greater than the within-individual effect for the latter). The ability to perform ADLs is associated with cognitive, motor, and perceptual functioning (34) and predicts mortality and morbidity (35–37). While we are not aware of any studies of IIV in cognitive performance and ADL functioning among older groups specifically, there have been studies of this association in other populations. For example, greater IIV across tasks has been found to predict poorer functioning in basic ADLs in human immunodeficiency virus (HIV)-seropositive individuals without HIV-associated neurocognitive disorders (38).

We also found that lower educational levels were associated with greater IIV. In a study of within-occasion reaction time IIV in cognitive tests, Christensen et al. (39) also found that participants with fewer years of education had, on average, greater IIV. Our results additionally indicated that lower educational levels predicted lower mean performance, mirroring the results of an earlier analysis of the same word recall test that used data from ELSA and the American Health and Retirement Study (40).

When interviewers indicated that there were issues which may have affected the cognitive tests, the probability of recalling words correctly was lower and IIV was greater. The between-individual effect was estimated to be greater than the within-individual effect, which may reflect the heterogeneity of potential issues, with these perhaps tending to vary more between people than within people. As Figure 5 indicates, these associations were relatively large, so test reliability is likely to be particularly low in such circumstances; thus, for researchers, it is crucial to record any difficulties and take this into account in analyses.

We found that people with a higher estimated probability of answering correctly at the sample mean age of 74.2 years had, on average, lower IIV. Indeed, greater IIV in cognitive functioning has been previously found to typically predict lower mean scores (7). This was also true at the population level for some of the covariates, but not all: For example, while females were estimated to have higher mean memory score than males, their IIV was, on average, greater. With regard to the association of sex with mean performance, this concurs with the findings of Zaninotto et al. (32), who also found females in the ELSA cohort to have a higher mean memory test score than males, with no moderating effect of age, although less explicit attention has been paid to the estimated effects of sex on IIV in memory-based tasks (cf. reaction-time tasks, where females are typically found to have greater IIV than males throughout adulthood (41–43)). Similarly, while later cohorts were estimated to have a higher probability of correctly recalling words, their IIV was estimated to be greater as well. This points to the utility of estimating the location (average probability, in our case) and scale (IIV) of repeatedly measured cognitive tests: estimating quantities which are, to an extent, orthogonal. We also found that persons estimated to have poorer episodic memory at the sample mean age of 74.2 years experienced, on average, a faster rate of decline in memory, concurring with a latent group analysis of this outcome in ELSA participants aged 65–79 years conducted by Olaya et al. (44). In addition, our results indicated that those whose mean performance declined less steeply had, on average, lower IIV. At the individual level, then, there tends to be a clustering together of higher mean performance, more gradual mean decline, and lower IIV and vice versa.

This study had several strengths. By employing a MELS model, we were able to investigate the associations of both time-varying and invariant factors with IIV, an opportunity that would be lost if instead we were deriving an individual-level summary statistic for IIV. A MELS model also adjusts for sampling variability (12), unlike methods which do not allow the within-individual sample size to inform the estimate of IIV. In addition, by using a beta-binomial model, we applied an analytical method appropriate to bounded discrete outcomes (17), avoiding bias in estimates of IIV due to floor/ceiling effects (18). Furthermore, the ELSA cohort was designed to be representative of the older-aged English population, and comparisons of sociodemographic data with data from the UK national census suggest that this is broadly the case (20).

This study also had limitations, however. There is the possibility of bias due to selection into the study. While ELSA was designed to be representative of the target population, there was attrition, as in any longitudinal cohort study, and we additionally did not observe the outcome for people responding by proxy (since the word recall test cannot be administered by proxy). If selection into our analysis depended only on variables included in our models, such as age, sex, education, difficulties with ADLs, interviewer-recorded issues with cognitive tests, cohort, and observed values of the outcome, our models will have been unbiased. Having conditioned on these covariates, if selection into our analysis depended on the outcome, however, there may have been bias. We also assumed that the number of observations was independent of the underlying risk of dropout (due to death, for example), which may otherwise lead to bias. While there has been limited research into the issue of missing data in MELS models, it is an important issue that is starting to receive attention (45), with a need for further work, including for non-Gaussian outcomes, where the computational burden of methods designed to ameliorate bias is likely to be considerable (46). Furthermore, people with a very modest number of observations will inform estimates of associations with IIV less than those observed more often (the same is true for the location, albeit to a lesser extent).

 In addition, while MELS models can be used in visit-to-visit designs such as this (15, 16), where longer time lags between waves typically mean much smaller within-person sample sizes than in data collection protocols which are more intensive (12), greater within-person sample sizes would improve the power with which IIV can be characterized. Furthermore, the trials comprising each outcome value are not strictly independent: A word recalled successfully in the immediate recall phase may be more likely to be recalled in the delayed recall phase (the sum of which constitutes the outcome), and memorizing word lists may involve mnemonic strategies which would violate the assumption of independence (47); the beta-binomial accommodates variation in dispersion, but more detailed research is needed to explore performance across different data-generating mechanisms. Indeed, while this total score is commonly analyzed as an indicator of episodic memory performance across a variety of cohorts (48–51), and some studies have found substantively similar findings between this and the constituent tests (52), the extent to which they may nevertheless map onto different memory systems is an area of ongoing investigation (53). In addition, our estimate of IIV depends on the fit of the model for the location (i.e., the linear predictor for average probability, *p*). The scientific significance of the modeled dispersion (IIV) parameter θ therefore depends on the choice of covariates in the fixed and random parts of the model for *p*, and also the extent of any measurement error they have. We chose covariates that were, a priori, of interest and tried to keep a balance between parsimony and detail, but nevertheless the dispersion parameter, θ, may have included variation which could be explained by a richer model for the location of the outcome. Finally, while a beta-binomial model can be more appropriate in certain circumstances, log(standard deviation) in a Gaussian model, for example, is on a more readily interpretable scale than log(θ) (although one might exponentiate the log scale to derive variance ratios (54)).

In summary, analyzing a memory test conducted with older people in ELSA, we found evidence of systematic differences in IIV, and also, having adjusted for those effects, evidence of residual between-individual differences in IIV. This indicates that sampling protocols for cognitive tests which rely on a single measurement occasion or a few measurement occasions to estimate mean group differences can be prone to considerable measurement error (7). At the population level, IIV in cognitive functioning provided information which was orthogonal to mean performance, emphasizing the importance of explicitly modeling IIV, rather than treating it as just a nuisance. In this study of visit-to-visit IIV, where measurements were made approximately every 2 years, inconsistency in task performance could be the result of both shorter-term (within-week, -day, -hour, etc.) and longer-term (e.g., over weeks or months) changes in mean performance levels. Study designs which repeatedly measure cognitive functioning over a variety of time frames (repeated “bursts” of measurement) would allow further characterization of IIV over the shorter and longer terms, which may differentially map onto underlying constructs of interest (13, 55, 56). Our study extended beta-binomial models which allow mixed effects for average probability (17, 57), to further include fixed and random effects for dispersion. We supply example software code with which to fit these MELS beta-binomial models via an efficient Bayesian engine (Stan via “brms” in R (29–31); see Web Appendix 2, and see Bonner et al. (58) for an alternative estimation method). While computational overheads are typically greater, and estimation can be more challenging, than with Gaussian models, the latter are not always appropriate—for example, for outcomes consisting of nonnegative integers with an upper bound. We applied this approach to aid our understanding of factors associated with IIV in cognitive functioning at older ages, providing insights into the mechanisms underlying differences in IIV and their role in predicting future outcomes, beyond mean performance (6, 7, 9). Future research is needed to investigate the impact of IIV on health and well-being.

## Supplementary Material

Web_Material_kwad169
