# Modelling risk factors for intraindividual variability: a mixed-effects beta-binomial model applied to cognitive function in older people in the English Longitudinal Study of Ageing

**DOI:** 10.1093/aje/kwad169

**Published:** 2024-01-08

**Authors:** Richard M.A. Parker, Kate Tilling, Graciela Muniz Terrera, Jessica K. Barrett

**Affiliations:** MRC Integrative Epidemiology Unit, University of Bristol, Oakfield House, Oakfield Grove, Bristol, BS8 2BN, United Kingdom; Population Health Sciences, Bristol Medical School, Oakfield House, Oakfield Grove, Bristol, BS8 2BN, United Kingdom; MRC Integrative Epidemiology Unit, University of Bristol, Oakfield House, Oakfield Grove, Bristol, BS8 2BN, United Kingdom; Population Health Sciences, Bristol Medical School, Oakfield House, Oakfield Grove, Bristol, BS8 2BN, United Kingdom; Department of Social Medicine, Ohio University, Athens, OH 45701, United States; Edinburgh Dementia Prevention, University of Edinburgh, Edinburgh, EH4 2XU, United Kingdom; >BrainLat, Universidad Adolfo Ibáñez, Santiago, Chile; University of Cambridge, MRC Biostatistics Unit, Cambridge, CB2 0SR, United Kingdom

**Keywords:** Heteroscedasticity, mixed effects model, beta binomial, intraindividual variability, cognitive test, older adults, Bayesian hierarchical model

## Abstract

Cognitive functioning in older age profoundly impacts quality of life and health. Whilst most research in cognition in older age has focussed on mean levels, intraindividual variability (IIV) around this may have risk factors and outcomes independent of the mean. Investigating risk factors associated with IIV has typically involved deriving a summary statistic for each person from residual error around a fitted mean. However, this ignores uncertainty in the estimates, prohibits exploring associations with time-varying factors, and is biased by floor/ceiling effects. To address this, we propose a mixed-effects location scale beta-binomial model to estimate average probability and IIV in a word recall test in the English Longitudinal Study of Aging. After adjusting for mean performance, an analysis of 9,873 individuals across 7 (mean: 3.4) waves (2002-2015), found IIV greater: at older ages; with lower education; in females; with more difficulties with activities of daily living; in later cohorts; and when interviewers recorded issues potentially affecting the tests. Our study introduces a novel method to identify groups with greater IIV in bounded discrete outcomes. Our findings have implications for daily functioning and care, with further work needed to identify the impact for future health outcomes.

Cognitive functioning in older people has profound implications for current and future health and wellbeing (1, 2). In the absence of therapeutical cures for dementia, for example, changes in cognitive performance can aid the early identification of individuals at increased risk of developing the condition, paramount for the design and implementation of interventions that may delay the onset of faster deterioration (3, 4). Traditionally, research in cognitive decline has focused on the study of individual differences and the identification of risk factors for rate of mean change (5). However, some have investigated inconsistency in performance (6, 7). For example, an analysis of the Sydney Memory and Ageing Study found that greater intraindividual variability (IIV), but not greater mean reaction time, significantly predicted survival time after adjusting for sociodemographic factors, cardiovascular risk and apolipoprotein ε4 status (8). Similarly, Gamaldo *et al*. investigated data from the Baltimore Longitudinal Study of Aging, showing that individuals who had received a diagnosis of dementia had greater variability in attention, executive function, language and semantic memory at least 5 years before the onset of cognitive impairment compared to individuals who remained free of dementia, demonstrating the potential role of IIV as an early indicator of pathological changes (9).

Despite the increasing interest in IIV, the analytical approaches commonly used to quantify it in longitudinal studies are limited. Some researchers have considered the average amount of deviation (residual error around a fitted mean) in an individual’s performance over time (10, 11). However this does not adjust for uncertainty in the estimates given the finite number of within-person observations, and the resulting individual-level summary statistic is not amenable to the exploration of associations of IIV with time-varying factors. Alternatively, Gamaldo *et al*. fitted multilevel models (MLMs) to repeated measurements of the outcome of interest, and then compared models which assume the residual IIV to be constant to models which allow it to depend on fixed effects, e.g. diagnostic status (9). However, this assumes that people within each group have the same IIV. It has been previously shown that in an MLM, the residual IIV can instead be assumed to contain systematic variation that can be explained, depending not only on fixed effects (as in Gamaldo *et al*. (9)), but on random effects as well, in mixed-effects location scale (MELS) models (12–16). MELS models allow for the association of IIV with predictors which may be time-varying, or otherwise, to be investigated. They further allow for residual differences between people in their IIV to be estimated via random effects, and their association with the individual mean to be investigated via correlated random effects (12).

Whilst MELS models typically assume that, conditional on the random effects, the response variable is Normally distributed, this is likely to be violated with a bounded discrete outcome, which are commonly used in the assessment of a variety of domains (17, 18). Here, floor and ceiling effects can lead to underestimated IIV for people returning high, or low, mean scores. Under such circumstances, a beta-binomial model has been shown to improve statistical inference (18). In contrast to some other approaches to analysing such data, including Gaussian, Poisson and negative binomial models, the beta-binomial supports non-negative integers with an upper bound: qualities found in many cognitive test outcomes. In addition, rather than directly modelling the probability of trial success, it models the distribution of probabilities. To investigate factors associated with IIV of cognitive performance we propose a model somewhat analogous to the MELS formulation, where the ‘location’ and ‘scale’ parameters of the beta-binomial model are each allowed to differ across fixed and random effects.

Since the evidence on factors associated with differences in IIV over time is limited, we use a MELS beta-binomial model to investigate visit-to-visit IIV in a word recall test in the English Longitudinal Study of Ageing (ELSA). Our aim is to introduce a modelling approach appropriate to this bounded discrete outcome to better understand the factors associated with IIV in a test of episodic memory in older adults.

## Methods

### Cohort

Participants were from the English Longitudinal Study of Ageing (ELSA) (19), an ongoing panel study that contains a nationally representative sample of the English population aged 50 and over living in households (20, 21). Interviews at baseline (2002–2003) were carried out with 11,391 individuals (5,186 men and 6,205 women); the overall response rate was 70% at the household level and 67% at the individual level. After the baseline interview, follow-up interviews took place at regular 2-year intervals in 2004–2005 (wave 2), 2006–2007 (wave 3), 2008–2009 (wave 4), 2010–2011 (wave 5), 2012–2013 (wave 6) and 2014-2015 (wave 7). Refresher samples were added at

waves 3, 4, 6 and 7 to ensure the study remained representative of the target age group. Participants gave full informed consent to participate in the study. We restricted our sample to core participants, and to observations made when aged 65 years old or older for which the participant did not report having Alzheimer’s disease or dementia, organic brain syndrome, senility or any other serious memory impairment.

### Cognitive function

Memory was measured using a 10-word recall test as earlier used in the Health and Retirement Study (22). Participants were presented with a list of 10 words that were read out to them and asked to recall as many words as they could both immediately and, with no prior notice, five minutes later and after they had been asked to complete other survey questions. The number of correctly recalled words was used as a measure of memory (range: 0–20 words) adding the results from both the immediate and delayed recall tests.

### Covariates

Information on participants’ age, sex, education, and difficulties with Activities of Daily Living (ADLs) were recorded at each wave. In addition, the interviewer reported whether there were any factors which may have impaired the participants’ performance during the cognitive tests. Age was centred (on the sample mean of 74.2) and in decades. Cohort was the year the participant turned 65, centred around 1999, also as decades. Education was categorised into higher (college / university), secondary and no qualifications. For difficulties with ADLs, participants were asked if they had any difficulty dressing (including putting on shoes and socks), eating (including cutting up food), bathing and showering, getting in and out of bed and walking across a room, and a count of the number of items participants had difficulties with was derived from this. Interviewer-recorded factors which may have affected the cognitive tests included: the participant being blind or having poor eyesight, being deaf or having poor hearing, being too tired, illness or physical impairment, impaired concentration, being very nervous or anxious, having other mental impairment, an interruption or distraction, a noisy environment, problems with the testing computer, difficulty in understanding English, or any other factors. The resulting variable took the value of zero if there were no such issues recorded, and one otherwise. Difficulties with ADLs, and interviewer-recorded factors potentially affecting tests, were both fitted as person-level means, to estimate their between- individual effect, and as person-level mean centred variables, to estimate their within-individual effect (23).

### Analytical approach

A mixed-effects location scale (MELS) beta-binomial model was fitted to repeated measurements of the longitudinal outcome, the word recall test score. The beta-binomial model assumes that each observed value of the outcome is the result of *n* Bernoulli trials which have an underlying, unobserved probability of success as sampled from a beta distribution (24, 25). The shape of the beta distribution from which these probabilities are drawn is defined by an average probability parameter *p* (a.k.a. *μ*; modelled on the logit scale) and a variability (or dispersion or variance) parameter *θ* (a.k.a. *φ*, *κ* or *ρ*; modelled on the log scale) (17, 26, 27).

**Figure 1:**
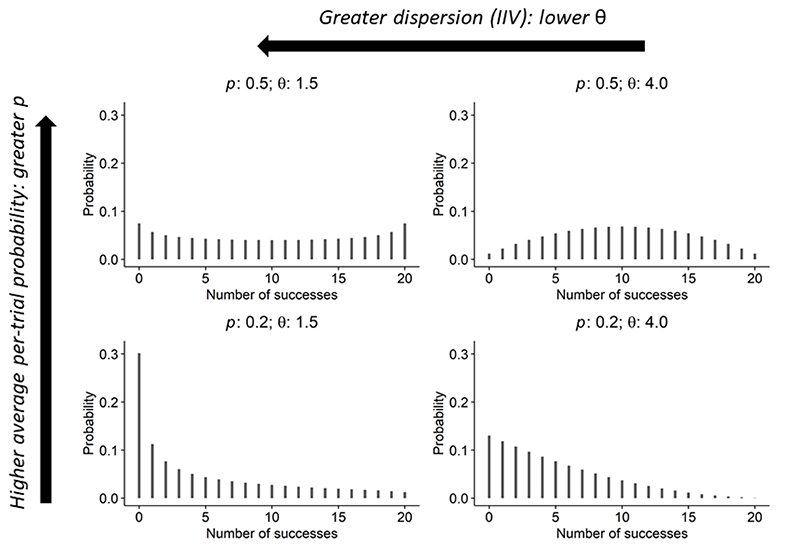
Mean probability of counts (from 20 trials) assuming beta distributions of underlying probabilities with different values of average probability (*p*) and dispersion (θ). E.g. when *p* = 0.5, if θ > 2 the distribution of probabilities is relatively concentrated around the mean, whilst if θ < 2 dispersion is greater and extreme probabilities near 0 and 1 are more likely than the mean (if θ is 2 (not shown), then every probability, from 0 to 1, would be equally-likely (26)). As this figure further illustrates, the value of θ is orthogonal *p*. See Supplementary Materials for additional examples.

Instead of directly modelling the probability for each observed count, the beta-binomial models this distribution of probabilities, via *p* and *θ*. Whilst a MELS model is typically a Gaussian model, we use the terminology here as we include both fixed (population) and random (individual) effects in (1) the linear predictor for *p* (the ‘location’ of the beta distribution), and (2) in the linear predictor for *θ* (the ‘scale’ of the beta distribution, where low estimated values of *θ* imply greater intraindividual variability (IIV) in task performance). Figure 1 plots expected distributions of test scores given illustrative values for these parameters.

Covariates added to the linear predictor for *p* were age, cohort (the year of reaching age 65), sex, educational qualification, the number of ADLs with which the respondent reported difficulty, and whether the interviewer reported whether there were any factors which may have impaired the participants’ performance during the cognitive tests. These covariates were also included in the linear predictor for *θ*. For the linear predictor for *p*, any non-linearity in the association between age and the outcome was first assessed by fitting restricted cubic regression splines with different sets of knots as recommended by Harrell (2015) (28), and the best-fitting function of age was selected and fitted. Interactions of age with each of sex, educational qualifications, number of ADL difficulties, and issues potentially impairing test performance were also added, in turn, to the linear predictor for *p* to see if they improved model fit. Model fit was assessed via Pareto smoothed importance sampling leave-one-out (PSIS-LOO) cross-validation (29). In addition, random effects were included in both the functions for *p* and *θ* to account for unobserved heterogeneity between individuals. In the predictor for *p*, a random intercept estimated the between-individual variability in *p* at the mean age, and a random slope estimated the between-individual variability of the effect of age (as a linear term fitted across the whole age range) on *p*. In the linear predictor for *θ*, a random intercept estimated the extent to which people differed in their IIV (specifically in how dispersed the beta distribution from which the underlying probability of test success was drawn; see Figure 1 for examples). Random effects were assumed multivariate normally distributed, allowing for non-zero correlations between them.

The models were fitted using Bayesian estimation via Markov chain Monte Carlo methods in Stan (2.21.0), using the brms package (2.16.1) in R (4.1.0) (30–32). Results are reported as means of posterior distributions and 95% credible intervals (CrI). See the Supplementary Materials for further details of these (and other sensitivity) analyses, including choice of priors, and sample code.

## Results

A total of 9,873 individuals were included in the final cohort (see Supplementary Figure S1). Of these, n = 2,202 (22.3%) were reported as dying during the study period, whilst the mortality status of 2,477 (25.1%) was reported as unknown at the final wave (wave 7), with the remaining 5,194 (52.6%) reported as alive at that wave. N = 396 (4.0%) of the final cohort were reported as having a memory problem in at least one survey (these, and subsequent, surveys for such participants were not included in the model). On average, participants included in the final cohort contributed data to 3.4 waves, with the number of observations per participant as follows, 1: 2,313; 2: 1,866; 3: 1,425; 4: 1,470; 5: 751; 6: 800; 7: 1,248.

Table 1 shows the baseline characteristics of included individuals, including by whether they contribute data to every wave after becoming eligible or not. It indicates that those who did not contribute data to every wave had, on average, a lower memory test score, were older, had lower educational qualifications, had more difficulties with ADLs, and had more issues recorded by the interviewer which may have affected the cognitive tests.

**Figure 2:**
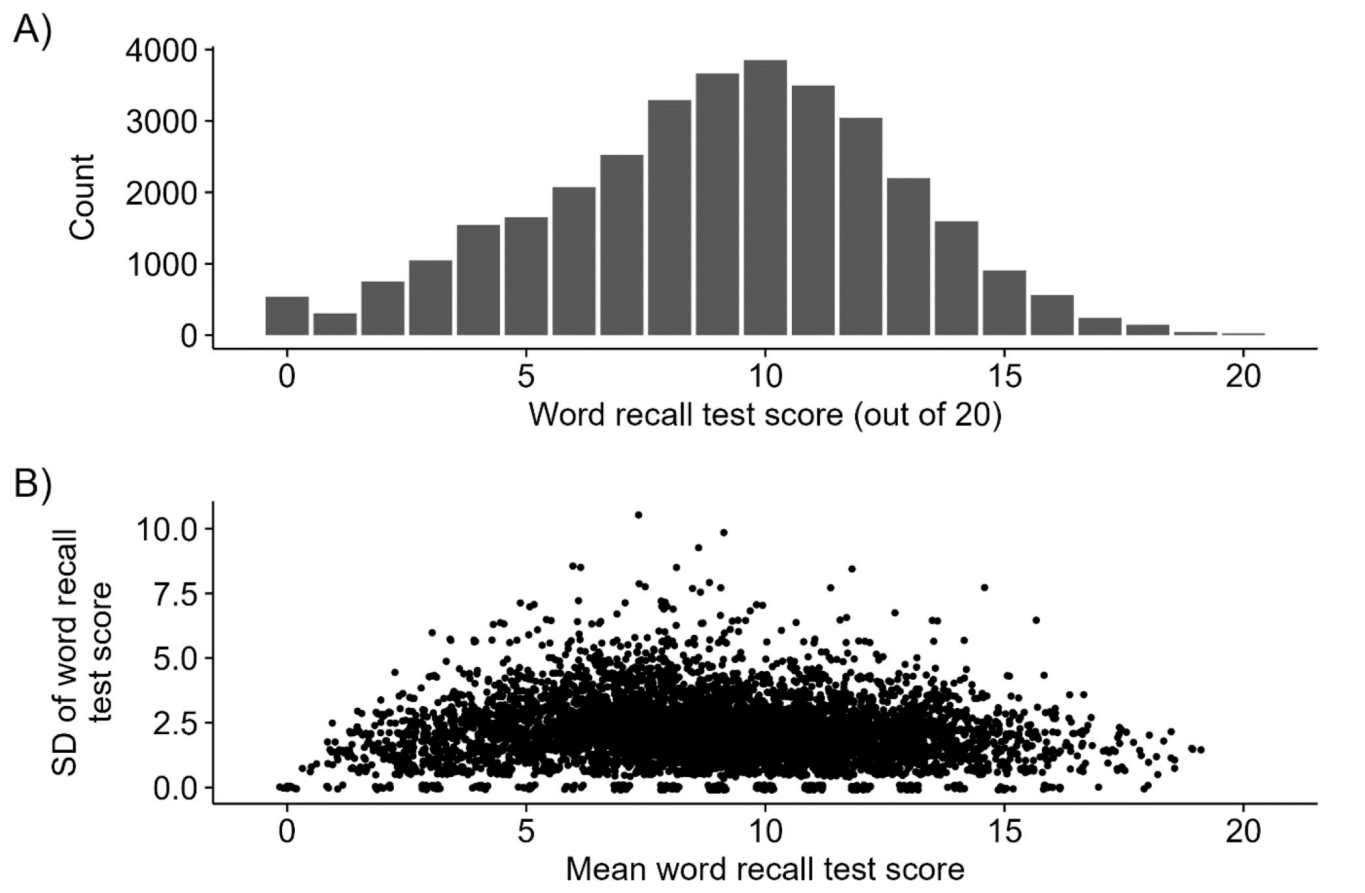
A) Bar chart of word recall test scores. B) Scatterplot of the standard deviation (SD) of the word recall test score against mean word recall test score, only plotting data from participants observed on 2 or more occasions (to calculate SD), and with a small amount of added jitter to avoid overplotting.

Figure 2 plots (A) the distribution, and (B) the mean vs. standard deviation (SD), of the word recall test, with the SD generally lower for people with high, or low, mean scores.

See Supplementary Materials for further summary statistics, including by exclusion status and drop-out status, and also for all estimates from the models presented below.

### Association of covariates with, *p* (average probability)

**Figure 3:**
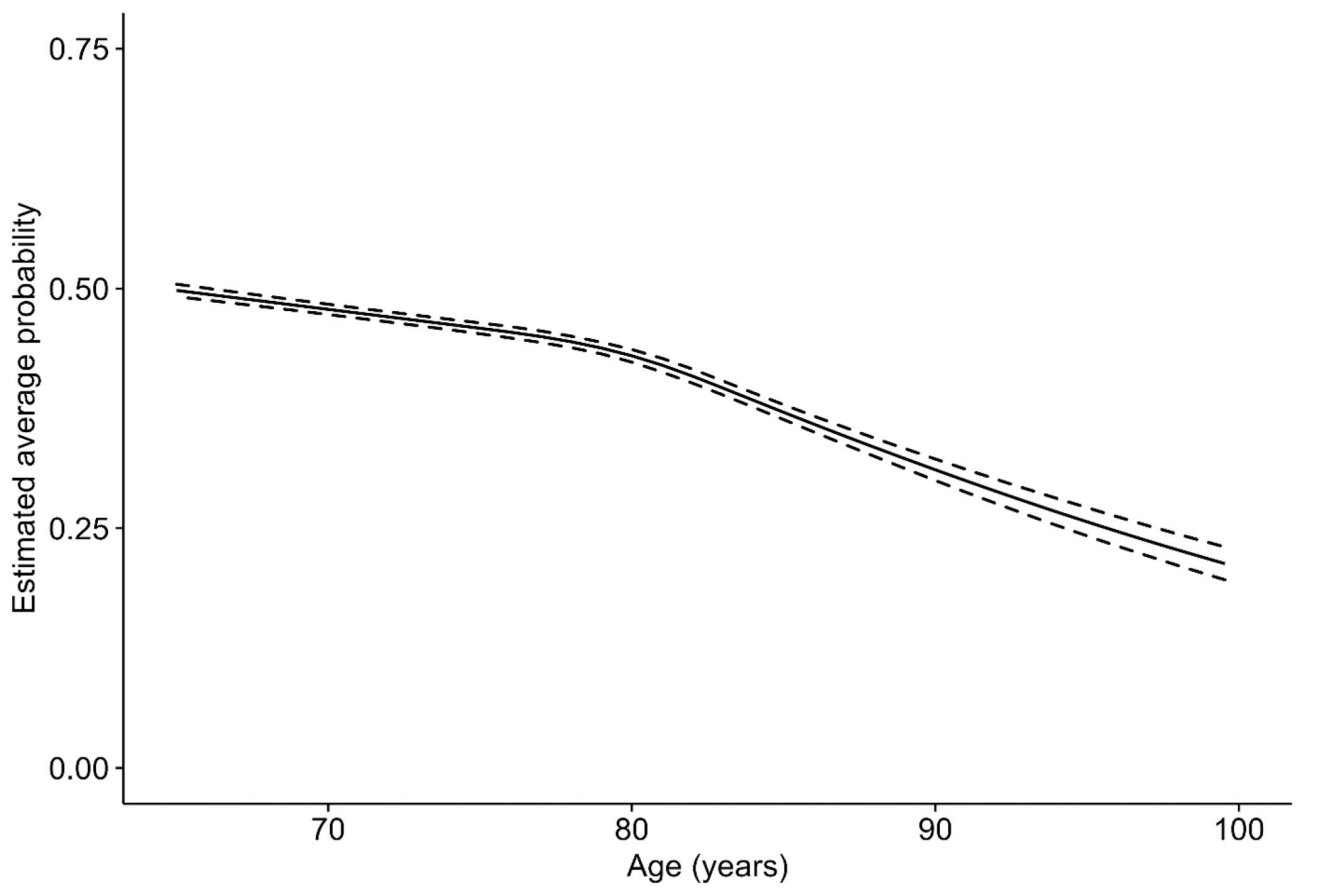
Predicted average probability (p) in the word recall test (with 95% Credible Interval) across age.

A restricted cubic spline for age, with 4 knots points, was fitted in the fixed part of the linear model for *p*; no interaction terms were selected (see Supplementary Materials). Figure 3 presents the estimated average probability of providing a correct answer in the word recall test, across age. It indicates that, on average, this probability decreased with age, with the rate of this decline greater from around 80 years of age.

**Figure 4:**
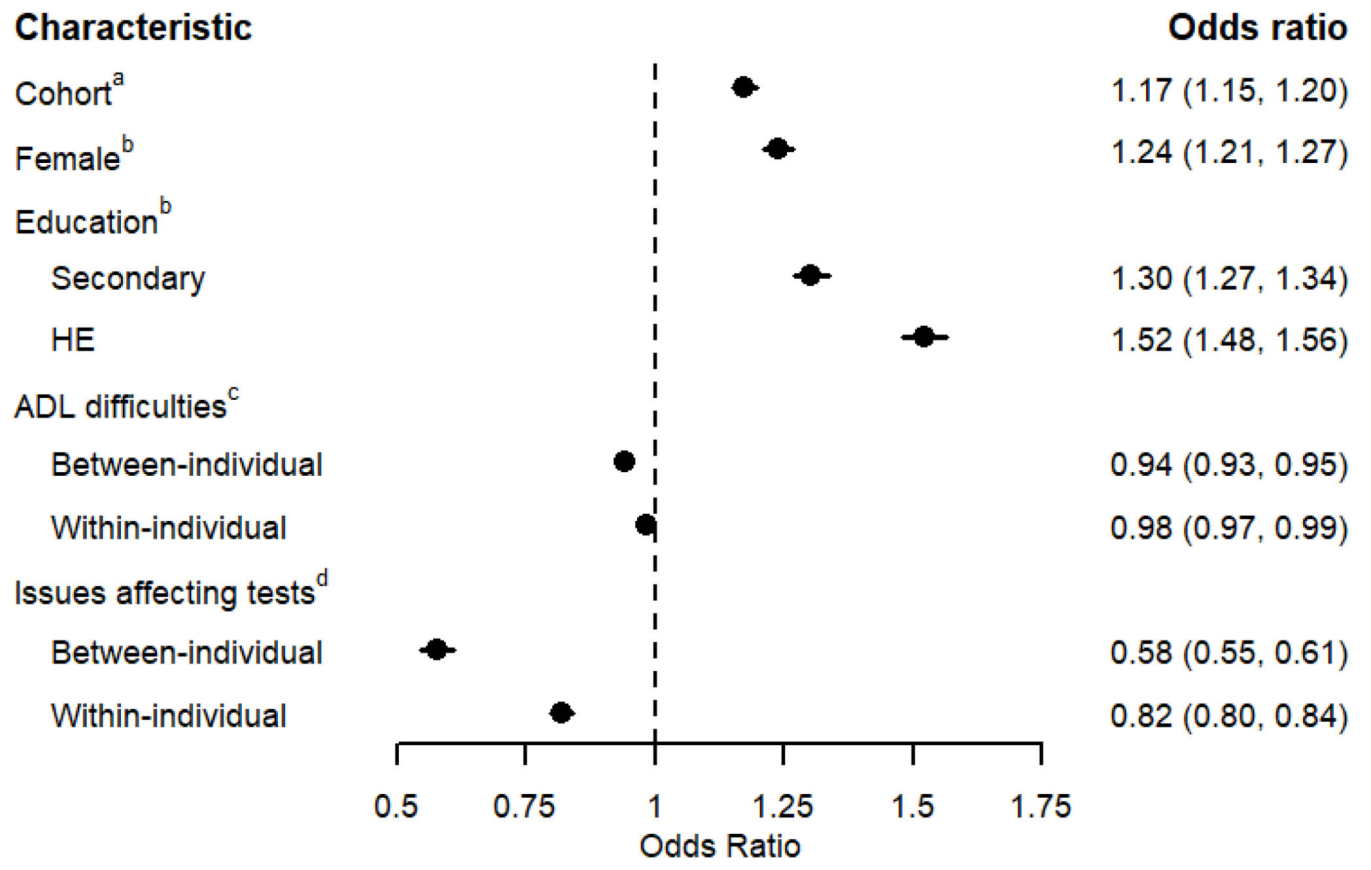
Estimated odds ratios (with 95% Credible Interval) for characteristics modelled in the linear predictor for average probability (*p*) in the word recall test. ^a^ The estimated average odds ratio for recalling a word correctly in the word recall test per 1 decade change in the year turned 65 (centred on 1999). ^b^ The estimated average odds ratio for recalling a word correctly in the word recall test when comparing the current category with the reference category. Reference categories as follows: Male (for Female); No qualifications (for Education). ^c^ The estimated average odds ratio for recalling a word correctly in the word recall test, for each additional difficulty reported with activities of daily living, fitted as a person-level mean, to estimate the between-individual effect, and also as a person-level mean centred variable, to estimates the within-individual effect (23). ^d^ The estimated average odds ratio for recalling a word correctly in the word recall test when comparing the current category (at least one interviewer-recorded factor which may have affected the test, coded as one) to the reference category (no such interviewer-recorded factors, coded as zero), fitted as a person-level mean, to estimate the between-individual effect, and also as a person-level mean centred variable, to estimates the within-individual effect (23).

Figure 4 presents the estimated odds ratios (OR) for the remaining covariates in the linear predictor for *p*. It indicates the probability of correctly recalling words declined, on average, as the number of difficulties with ADLs increased, with an OR for the between-individual effect of 0.94 (95% CrI: 0.93, 0.95) indicating a 6% lower odds of recalling a word correctly for each activity reported to be performed with difficulty, and an OR for the within-individual effect of 0.98 (95% CrI: 0.97, 0.99). Mean test score was lower when the interviewer recorded issues potentially affecting the test, with an OR for the between-individual effect of 0.58 (95% CrI: 0.55, 0.61), and an OR of 0.82 (95% CrI: 0.80, 0.84) for the within-individual effect. An estimated OR of 1.24 (95% CrI: 1.21, 1.27) indicated that the odds of recalling a word correctly were 24% higher for females than males. The odds were also higher in those with a higher level of education: compared to no educational qualifications, those with secondary educational qualifications had an OR of 1.30 (95% CrI: 1.27, 1.34), and those with HE qualifications had an OR of 1.52 (95% CrI: 1.48, 1.56). Finally, the odds were higher in later cohorts, with an OR of 1.17 (95% CrI: 1.15, 1.20) per decade later participants were born.

### Association of covariates with *θ* (IIV)

**Figure 5:**
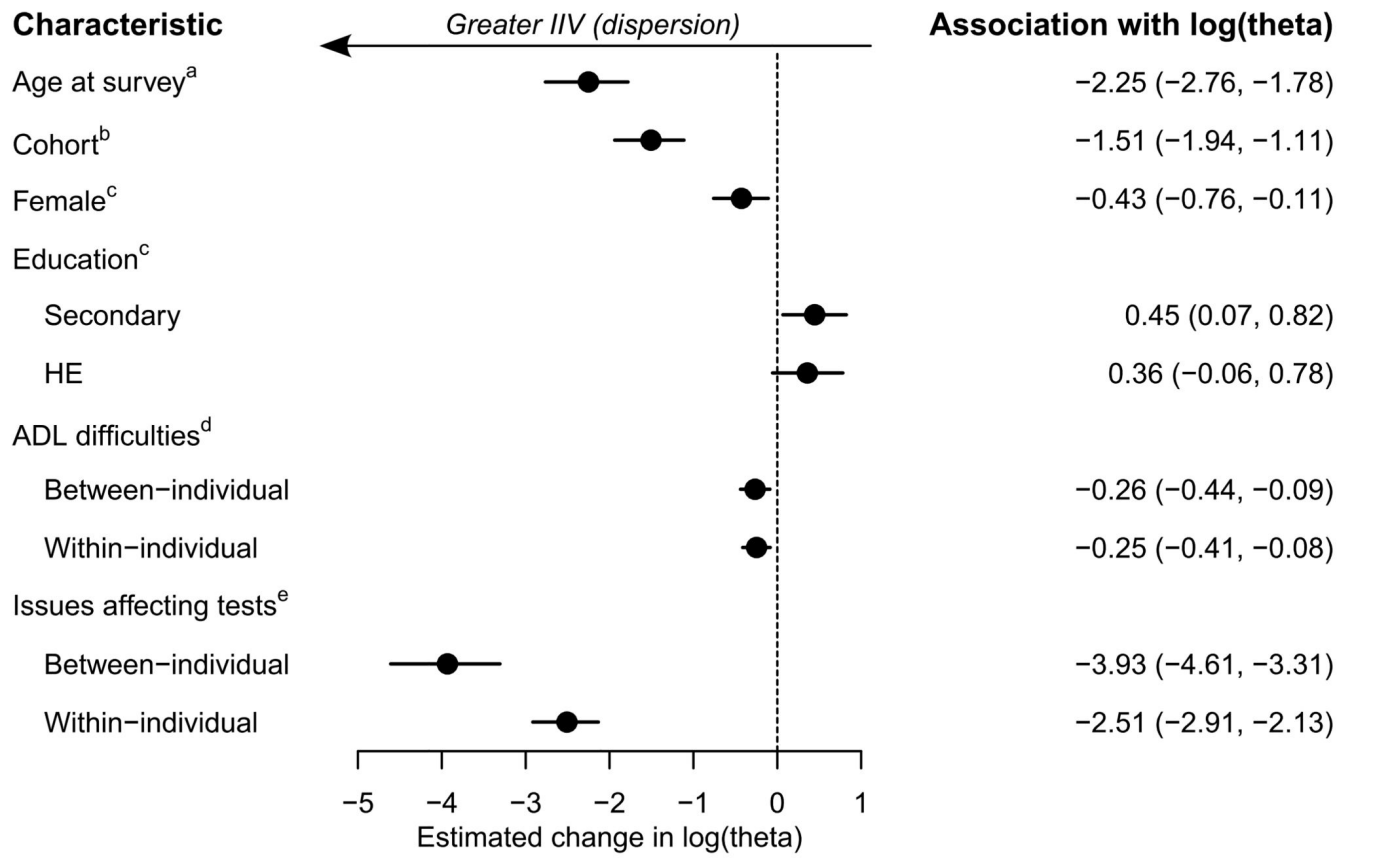
Estimated associations (with 95% Credible Interval) of modelled characteristics with IIV parameter (*θ*; theta) in the word recall test. Note that smaller values of log(*θ*) indicate greater dispersion (IIV). ^a^ The estimated change in log(*θ*) per 1 decade change in age (centred on the sample mean age of 74.2 years). ^b^ The estimated change in log(*θ*) per 1 decade change in the year turned 65 (centred on 1999). ^c^ The estimated change in log(*θ*) when comparing the current category with the reference category. Reference categories as follows: Male (for Female); No qualifications (for Education). ^d^ The estimated change in log(*θ*) for each additional difficulty reported with activities of daily living, fitted as a person-level mean, to estimate the between-individual effect, and also as a person-level mean centred variable, to estimates the within-individual effect (23). ^e^ The estimated change in log(*θ*) when comparing the current category (at least one interviewer-recorded factor which may have affected the test, coded as one) to the reference category (no such interviewer-recorded factors, coded as zero), fitted as a person-level mean, to estimate the between-individual effect, and also as a person-level mean centred variable, to estimates the within-individual effect (23).

Figure 5 presents the estimated change in the log of the intraindividual variability (IIV) or dispersion parameter, *θ*, for each modelled characteristic (NB lower values of *θ* indicate greater IIV: e.g. Figure 1). It indicates that older people had greater IIV in memory scores, on average, with log(*θ*) estimated to be to be -2.25 (95% CrI: -2.76, -1.78) lower for each decade increase in age at survey. As Figure 5 also illustrates, higher educational qualifications were associated with lower IIV, with those who completed secondary school, for instance, having an estimated log(*θ*) higher by 0.45 (95% CrI: 0.07, 0.82), compared to individuals without qualifications. IIV was greater for individuals with more difficulties in their ADLs, with the between-individual effect estimated to reduce log(*θ*) by an average of -0.26 (95% CrI: -0.44, -0.09) for each activity performed with difficulty, with a similar estimate for the within-individual effect (-0.25 (95% CrI: -0.41, -0.08)). IIV was higher for females, with log(*θ*) lower by an average of -0.43 (95% CrI: -0.76, -0.11) compared to males. When the interviewer had recorded issues which might have affected the tests, the IIV was higher, with log(*θ*) reducing by an average of -3.93 (95% CrI: -4.61, -3.31), for the between-individual effect, and -2.51 (95% CrI: -2.91, -2.13) for the within-individual effect. Later cohorts were also estimated to have greater IIV, with log(*θ*) lower by -1.51 (95% CrI: -1.94, -1.11) for each decade later participants were born.

### Participant effects

The random part of the model indicated that the correlation between average probability (*p*) at 74.2 years of age (random intercept) and rate of change in *p* (random slope) was estimated at 0.37 (95% CrI: 0.29, 0.47), suggesting that individuals with poorer episodic memory at age 74.2 years of age experienced a faster rate of decline in memory. Those who tended to score higher in the memory test (more positive random intercept for *p*) tended to have lower IIV (higher log(*θ*)), with a posterior mean correlation of 0.45 (95% CrI: 0.37, 0.52) between the random intercept (at 74.2 years of age) for *p* and the random intercept of the predictor for log(*θ*). Finally, the posterior mean correlation between the random slope for *p* and the random intercept for IIV (log(*θ*)) was 0.31 (95% CrI: 0.11, 0.51), indicating that those with slopes which decline less steeply (i.e. more positive estimates for the random slope) tended to have lower IIV (i.e. a higher estimate for log(*θ*)).

## Discussion

We have proposed and applied a mixed-effects location scale (MELS) beta-binomial model to simultaneously estimate average probability and intraindividual variability (IIV) in a discrete outcome, estimating fixed (population) and random (individual) effects for each. We found that IIV in a visit-to-visit test of episodic memory in older adults participating in ELSA changed across age, sex, education, the number of difficulties with activities of daily living (ADLs), cohort, and reported challenges when performing the test. We also found that people with a higher probability of recalling words correctly tended to have lower IIV, and those with a more gradual decline in mean performance over time also tended to have lower IIV.

Our finding that IIV in the word recall test increased with age, whilst the probability of correctly recalling words decreased with age, is characteristic of studies of cognitive functioning in advanced years, as reviewed in MacDonald *et al*., for example, who further discuss potential neural mechanisms underlying these age-related changes in IIV (7). In addition, earlier analyses of the ELSA cohort have similarly found a faster average rate of decline in average word recall test performance at older ages (33, 34). Higher mean memory performance in later cohorts, as we found, has also previously been reported in ELSA (34), although we additionally found IIV to be greater in later cohorts too.

Our results also indicated that the greater the number of difficulties participants had with ADLs, the greater their IIV in memory test performance, and the lower their probability of correctly recalling words too (with the between-individual effect greater than the within-individual effect for the latter). The ability to perform ADLs is associated with cognitive, motor and perceptual functioning (35), and predicts mortality and morbidity (36–38). Whilst we are not aware of any studies of IIV in cognitive performance and ADL functioning in older groups specifically, there have been studies of this association in other populations. For example, greater IIV across tasks has been found to predict poorer functioning in basic ADLs in HIV-seropositive individuals without HIV-associated neurocognitive disorders (39).

We also found that lower educational levels were associated with greater IIV. In a study of within-occasion reaction time IIV in cognitive tests, Christensen *et al*. also found participants with fewer years of education had, on average, greater IIV (40). Our results additionally indicated that lower educational levels predicted lower mean performance, mirroring the results of an earlier analysis of the same word recall test, using ELSA and the American Health and Retirement Study (41).

When interviewers indicated there were issues which may have affected the cognitive tests, then the probability of recalling words correctly was lower, and IIV was greater. The between-individual effect was estimated to be greater than the within-individual effect, which may reflect the heterogeneity of potential issues, with these perhaps tending to vary more between- than within-people. As Figure 5 indicates, these associations were relatively large, and so test reliability is likely to be particularly low in such circumstances, thus for researchers it is crucial to record any difficulties and take this into account in analyses.

We found people with a higher estimated probability of answering correctly at the sample mean age of 74.2 years had, on average, lower IIV. Indeed, greater IIV in cognitive functioning has been previously found to typically predict lower mean scores (7). This was also true at the population level for some of the covariates, but not all: for example, whilst females were estimated to have higher mean memory score than males, their IIV was, on average, greater. With regard to the association of sex with mean performance, this concurs with Zaninotto *et al*., who also found females in the ELSA cohort to have a higher mean memory test score than males, with no moderating effect of age (33), although less explicit attention has been paid to the estimated effects of sex on IIV in memory-based tasks (*cf*. reaction time tasks, where females typically found to have greater IIV than males throughout adulthood (42–44)). Similarly, whilst later cohorts were estimated to have a higher probability of correctly recalling words, their IIV was estimated to be greater too. This points to the utility of estimating the location (average probability, in our case) and scale (IIV) of repeatedly-measured cognitive tests: estimating quantities which are, to an extent, orthogonal. We also found that those estimated to have poorer episodic memory at the sample mean age of 74.2 years experienced, on average, a faster rate of decline in memory, concurring with a latent group analysis of this outcome in ELSA participants aged 65-79 conducted by Olaya *et al*. (45). In addition, our results indicated that those whose mean performance declined less steeply had, on average, lower IIV. At the individual-level, then, there tends to be a clustering together of higher mean performance, more gradual mean decline and lower IIV, and *vice versa*.

This study has several strengths. By employing a MELS model, we were able to investigate the association of both time-varying and invariant factors with IIV, an opportunity lost if instead deriving an individual-level summary statistic for IIV. A MELS model also adjusts for sampling variability (12), unlike methods which do not allow the within-individual sample size to inform the estimate of IIV. In addition, by using a beta-binomial model we have applied an analytical method appropriate to bounded discrete outcomes (17), avoiding bias in estimates of IIV due to floor/ceiling effects (18). Also, the ELSA cohort is designed to be representative of the older-aged English population, with comparisons of sociodemographic data with that from the national census suggesting this is broadly the case (21).

This study also has limitations, however. There is the possibility of bias due to selection into the study. Whilst ELSA is designed to be representative of the target population, as in any longitudinal cohort there is attrition, and we additionally do not observe the outcome for people responding by proxy (since the word recall test cannot be administered by proxy). If selection into our analysis depends only on variables included in our models, such as age, sex, education, difficulties with ADLs, interviewer-recorded issues with cognitive tests, cohort, and observed values of the outcome, then our models will be unbiased. Having conditioned on these covariates, if selection into our analysis depends on the outcome, however, then there may be bias. Furthermore, we assume that the number of observations is independent of the underlying risk of drop-out (due to death, for example), which may otherwise lead to bias. Whilst there has been limited research into the issue of missing data in MELS models, it is an important issue starting to receive attention (46), with the need for further work, including for non-Gaussian outcomes, where the computational burden of methods designed to ameliorate bias is likely to be considerable (47). In addition, whilst MELS models can be used in visit-to-visit designs such as this (15, 16), where longer time lags between waves typically mean much smaller within-person sample sizes than data collection protocols which are more intensive (12), greater within-person sample sizes would improve the power with which IIV can be characterised. Furthermore, people with a very modest number of observations will inform estimates of associations of IIV less than those observed more often (the same is true for the location, albeit to a lesser extent). In addition, the trials comprising each outcome value are not strictly independent: a word recalled successfully in the immediate recall phase may be more likely to be recalled in the delayed recall phase (the sum of which constitutes the outcome), and memorizing word lists may involve mnemonic strategies which would violate the assumption of independence (48); the beta-binomial accommodates variation in dispersion, but more detailed research is needed to explore performance across different data-generating mechanisms. Indeed, whilst this total score is commonly analysed as an indicator of episodic memory performance across a variety of cohorts (49–52), and some studies have found substantively similar findings between this and the constituent tests (53), the extent to which they may nevertheless map onto different memory systems is an area of ongoing investigation (54). Furthermore, our estimate of IIV depends on the fit of the model for the location (i.e. the linear predictor for average probability, *p*). The scientific significance of the modelled dispersion (IIV) parameter *θ* therefore depends on the choice of covariates in the fixed and random part of the model for *p*, and also the extent of any measurement error they have. We have chosen covariates which are, *a priori*, of interest, and have tried to keep a balance between parsimony and detail, but nevertheless the dispersion parameter, *θ*, may include variation which could be explained by a richer model for the location of the outcome. Finally, whilst a beta-binomial model can be more appropriate in certain circumstances, log(SD) in a Gaussian model, for example, is on a more readily-interpretable scale than log(*θ)* (although one might exponentiate the log scale to derive variance ratios (55)).

In summary, analysing a memory test conducted with older people in ELSA, we found evidence of systematic differences in IIV, and also, having adjusted for those effects, evidence of residual between-individual differences in IIV. This indicates that sampling protocols for cognitive tests which rely on one, or a few, measurement occasions to estimate mean groups differences can be prone to considerable measurement error (7). At the population-level, IIV in cognitive functioning provided information which was orthogonal to mean performance, emphasising the importance of explicitly modelling IIV, rather than treating it as just a nuisance. In this study of visit-to-visit IIV, where measurements were made approximately every two years, inconsistency in task performance could be the result of both shorter (e.g. within-week, day, hour, etc.), and longer (e.g. over weeks or months), term changes in mean performance levels. Study designs which repeatedly-measure cognitive functioning over a variety of timeframes (repeated ‘bursts’ of measurement) would allow further characterisation of IIV over the shorter and longer term, which may differentially map onto underlying constructs of interest (13, 56, 57). Our study extends beta-binomial models which allow mixed effects for average probability (17, 58), to further include fixed and random effects for dispersion. We supply example code to fit these MELS beta-binomial models via an efficient Bayesian engine (Stan via brms in R (30–32); see also Bonner *et al*. (59)). Whilst computational overheads are typically greater, and estimation can be more challenging, than Gaussian models, the latter are not always appropriate, for example for outcomes consisting of non-negative integers with an upper bound. We apply this approach to aid our understanding of factors associated with IIV in cognitive functioning in older ages, providing insights into the mechanisms underlying differences in IIV, and their role in predicting future outcomes, beyond mean performance (6, 7, 9). Future research is needed to investigate the impact of IIV on health and wellbeing.

## Supplementary Material

Supplementary File

## Figures and Tables

**Table 1:** Summary statistics at baseline for individuals included in the model, further subdivided by whether they contribute data to every wave after becoming eligible for inclusion or not.

Characteristic at baseline:^a^ Mean (SD) or Number of individuals (%)	Included in model (n = 9,873)	Included in model but do not contribute data to every wave after becoming eligible for inclusion (n = 5,421)	Included in model and contribute data to every wave after becoming eligible for inclusion (n = 4,452)
Age^b^	70.5 (6.4)	73.1 (7.0)	67.3 (3.5)
Cohort, year turned 65			
1971-1980	282 (2.9%)	278 (5.1%)	4 (0.1%)
1981-1990	1595 (16.2%)	1444 (26.6%)	151 (3.4%)
1991-2000	3058 (31.0%)	2098 (38.7%)	960 (21.6%)
2001-2010	3574 (36.2%)	1488 (27.4%)	2086 (46.9%)
2011-2020	1364 (13.8%)	113 (2.1%)	1251 (28.1%)
Sex			
Male	4490 (45.5%)	2512 (46.3%)	1978 (44.4%)
Female	5383 (54.5%)	2909 (53.7%)	2474 (55.6%)
Education			
None	4731 (47.9%)	3223 (59.5%)	1508 (33.9%)
Secondary	2849 (28.9%)	1327 (24.5%)	1522 (34.2%)
Higher education	2293 (23.2%)	871 (16.1%)	1422 (31.9%)
Number of activities of daily living performed with difficulty^c^			
0	7723 (78.2%)	3912 (72.2%)	3811 (85.6%)
1	1158 (11.7%)	783 (14.4%)	375 (8.4%)
2	521 (5.3%)	361 (6.7%)	160 (3.6%)
3	292 (3.0%)	216 (4.0%)	76 (1.7%)
4	144 (1.5%)	120 (2.2%)	24 (0.5%)
5	35 (0.4%)	29 (0.5%)	6 (0.1%)
Issues with cognitive tests^d^			
No	8634 (87.5%)	4515 (83.3%)	4119 (92.5%)
Yes	1239 (12.5%)	906 (16.7%)	333 (7.5%)
Word recall test	9.2 (3.7)	7.9 (3.6)	10.7 (3.2)

a Where baseline is first wave included in the model for each individual.

b Note that this differs from the sample mean quoted in the main text, as it is calculated from the baseline observations only, rather than from all the repeated measurements.

c Sum of the activities of daily living (ADLs) with which participant reported any difficulty. ADLs include bathing, dressing, eating, getting in/out of bed, walking across a room.

d Interviewers recorded any issues which may have affected the participant’s performance in the cognitive tests.

## Data Availability

The English Longitudinal Study of Ageing (ELSA) dataset is available to UK Data Service registered users subject to an End User Licence Agreement (http://www.ukdataservice.ac.uk; DOI: 10.5255/UKDA-SN-5050-24).

## Abbreviations

ADLs: Activities of Daily Living
CrI: credible interval
ELSA: English Longitudinal Study of Aging
IIV: intraindividual variability
MELS: mixed-effects location scale
MLM: multilevel model
OR: odds ratio
SD: standard deviation
